# Complete Genome Sequence of *Rothia mucilaginosa* Strain NUM-Rm6536, Isolated from a Human Oral Cavity

**DOI:** 10.1128/genomeA.01122-15

**Published:** 2015-10-01

**Authors:** Takayuki Nambu, Osamu Tsuzukibashi, Satoshi Uchibori, Chiho Mashimo

**Affiliations:** a Department of Bacteriology, Osaka Dental University, Hirakata, Osaka, Japan; b Department of Oral Microbiology, Nihon University School of Dentistry at Matsudo, Chiba, Japan; c Department of Crown Bridge Prosthodontics, Nihon University School of Dentistry at Matsudo, Chiba, Japan

## Abstract

Here, we present the complete genome sequence of *Rothia mucilaginosa* NUM-Rm6536, a strain isolated from the tongue plaque of a healthy human adult. This strain is amenable to genetic manipulation by transformation and so provides a useful foundation for more detailed investigation of this species.

## GENOME ANNOUNCEMENT

*Rothia mucilaginosa*, previously known as *Stomatococcus mucilaginosus*, is a resident of the human oral cavity and upper respiratory tract (1). Although several studies have suggested that *R. mucilaginosa* comprises one member of a healthy oral microbiota (2–4), there have been an increasing number of reports on infections caused by this organism, especially among immunocompromised patients (5, 6). More recently, this organism was reported as a species isolated from sputum of cystic fibrosis (CF) patients and defined as a new emerging CF pathogen (7). We previously determined the complete genomic sequence of *R. mucilaginosa* DY-18, a clinical isolate from a persistent apical periodontitis lesion (DDBJ/EMBL/GenBank accession no. AP011540) (8). The genome of DY-18 encodes two sigma factors, one of which is an extracytoplasmic function (ECF) sigma factor markedly up-regulated under disulfide stress (9), but this strain is not amenable to genetic transformation. Therefore, we isolated a transformable strain of *R. mucilaginosa* (NUM-Rm6536) from tongue plaque of a healthy human adult (our unpublished data) (10). The aim of the present study is to convey the full genome sequence of NUM-Rm6536.

Total bacterial DNA of NUM-Rm6536 was extracted from an overnight culture using a Nucleo Spin Tissue kit (Macherey-Nagel). A 20-kb SMRTbell library was prepared, and the genome was sequenced using a PacBio RS II system (Pacific Biosciences) on a single-molecule real-time (SMRT) cell using PacBio P6-C4 chemistry.

*De novo* assembly of 129,310 reads with a mean length of 6,659 bp using the hierarchical genome assembly process (HGAP) algorithm in SMRT Analysis software version 2.3 (11) revealed a closed circular chromosome 2,292,716-bp in size with average coverage of 222.27×. The genome has a G+C content of 59.56%. The genome was then annotated using RAST (Rapid Annotation using Subsystem Technology) software version 2.0 (12), which successfully identified 1,762 coding sequences, as well as 59 RNA sequences. Of these, 45% of the annotated coding sequences fell within 280 subsystems available in the RAST database. The annotated data set presented here should augment future study of this organism in addition to providing resources for genetic manipulation. 

### Nucleotide sequence accession number. 

The genome sequence of *Rothia mucilaginosa* strain NUM-Rm6536 has been deposited in the DDBJ/EMBL/GenBank database under accession number AP014938.
